# Attending Over Triads for Learning Signed Network Embedding

**DOI:** 10.3389/fdata.2019.00006

**Published:** 2019-06-06

**Authors:** Shagun Sodhani, Meng Qu, Jian Tang

**Affiliations:** ^1^Département d'informatique et de Recherche Opérationnelle, Montreal Institute for Learning Algorithm, Université de Montréal, Montreal, QC, Canada; ^2^HEC, Université de Montréal, Montreal, QC, Canada

**Keywords:** signed network, network representation, structural balance theory, attention mechanism, higher order structures

## Abstract

Network embedding, which aims at learning distributed representations for nodes in networks, is a critical task with wide downstream applications. Most existing studies focus on networks with a single type of edges, whereas in many cases, the edges of networks can be derived from two opposite relationships, yielding signed networks. This paper studies network embedding for the signed network, and a novel approach called **TEA** is proposed. Similar to existing methods, **TEA** (**T**riad+**E**dge+**A**ttention) learns node representations by predicting the sign of each edge in the network. However, many existing methods only consider the local structural information (i.e., the representations of nodes in an edge) for prediction, which can be biased especially for sparse networks. By contrast, **TEA** seeks to leverage the high-order structures by drawing inspirations from the Structural Balance Theory. More specifically, for an edge linking two nodes, **TEA** predicts the edge sign by considering the triangles connecting the two nodes as features. Meanwhile, an attention mechanism is proposed, which assigns different weights to the different triangles before aggregating their predictions for more precise results. We conduct experiments on several real-world signed networks, and the results prove the effectiveness of **TEA** over many strong baseline approaches.

## 1. Introduction

Networks are the universal choice of data structure for representing relationships between interconnected objects in a wide variety of disciplines. This includes computer networks, biological network, chemical compounds, the socio-economical phenomenon like the six degrees of separation (Backstrom et al., 2011) etc. Given the ubiquity of network data, various approaches have been proposed in the literature to learn network embedding (Perozzi et al., 2014; Tang et al., 2015; Grover and Leskovec, 2016; Wang et al., 2016; Yang et al., 2016). These techniques map the nodes in the network to low dimensional real-valued vectors which can encode important node attributes and connectivity structure. Essentially, network embedding techniques aim to learn node embeddings which preserve proximity between the nodes. Such techniques are critical tools in the toolbox of network analysis. The learned node embeddings have been shown to be useful in a variety of downstream applications, such as node classification (Perozzi et al., 2014), link prediction (Grover and Leskovec, 2016) and graph visualization (Tang et al., 2016). The basic assumption underlying these methods is that the nodes, that are connected by edges, are more likely to be similar to each other than the unconnected nodes. This is known as the *homophily* effect (can be described as *Birds of a feather flock together*).

Though these network embedding techniques have been very successful on a variety of tasks and domains, they have largely focused on the unsigned networks. In the unsigned networks, all the edges are derived from similar semantic relationships like friendship, collaboration etc. While the edges can encode different relationships, the relationships themselves are not opposing in nature. But in many real-life situations, the interactions are not limited to positive interactions and the effect of negative (or opposing) relationships inter-plays with the positive effects. In the context of social networks, the interaction between the users could be friendly or hostile or the relationships could be based on trust or distrust. In such cases, the edges are derived from two opposite relationships and can have opposite signs, thus forming a signed network. For example, in the Epinions social network^1^, users can create relationships (links) with other users that are based on opposing semantics of “trust” (positive) and “distrust” (negative). In the Slashdot social network^2^, users can mark other users as “friends” and “foes,” forming the positive and the negative edges, respectively. Another instance could be in the context of elections where a voter could either vote in favor of (positive edge) or against (negative edge) a candidate. While the positive edges indicate strong relationship connections between nodes, the negative edges usually indicate anti-relationship connections. There is a subtle difference between negative edge and absence of an edge. The absence of a connection between two users in a social network does not tell us if the two users are likely to be friends or enemies while the presence of a negative edge tells us that the two users are enemies. Hence the effect of negative edges is not the same as the effect of absent positive edges. Further, the socio-psychological effects underlying the dynamics in signed networks are different from those for unsigned networks (section 2.2). Therefore, existing network embedding techniques, that have been developed in the context of unsigned networks, can not be applied directly on the signed networks.

It is important to highlight the difference between signed edges and labeled edges. In the case of labeled edges, the different type of relations may occur between different nodes but these relationships need not be contrasting or opposing. In the case of signed networks, the positive and the negative edges represent relationships that are opposite of each other-friend vs. foes, trust vs. distrust. The interplay of opposing relationships is distinct to the signed networks.

To learn effective node representations in the signed networks, some recent methods (Wang et al., 2017a,b; Wang H. et al., 2017) are proposed, which can take into account both the positive and the negative edges when learning the node representations. For example, Wang et al. (2017b) proposed an approach to discriminate between the positive edges, the negative edges and the unlabeled edge with a neural network classifier. Though their approach performs quite well on the link prediction task, they typically consider only the local structure of the given network. In other words, only the observed edges (i.e., positive and negative edges) are used during training. However, in many real-world networks, the observed edges can be very scarce, making existing methods perform poorly on such real-world networks. To learn more effective node representations, we seek to develop an approach that can leverage high-order network structures along with the observed edges when learning the representation of the given network.

**Figure 1:**
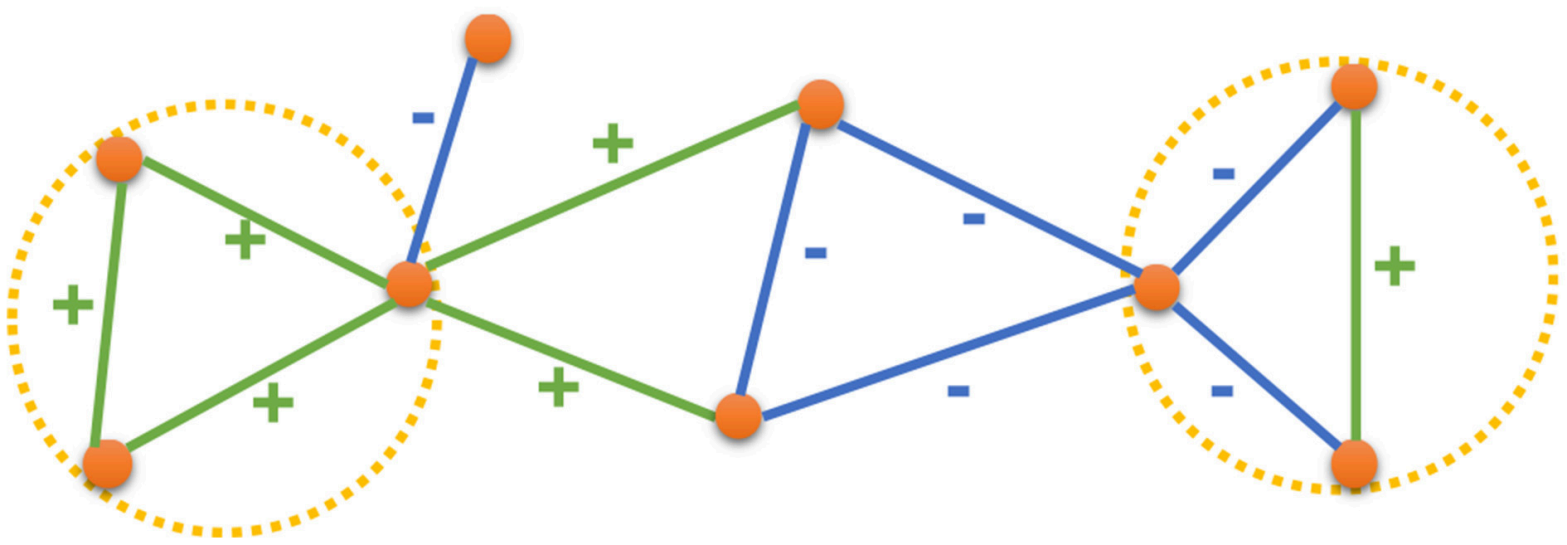
An example signed network, where green edges have positive signs and blue edges have negative signs. Yellow circles mark two examples of balance states.

In this paper, we leverage the high-order structures in the network by drawing inspiration from the Structural Balance Theory as described in the context of social networks and social psychology by Heider (1946) and as described in the context of graphs by Harary (1953), Davis (1963), and Cartwright and Harary (1956). The Structural Balance Theory states that in a social setup, people would prefer forming relationships such that a balanced state emerges in their interpersonal relationships. For example, consider a group of three nodes (users) where there exists an edge between all pair of nodes (that is, all the users have a relationship with each other). When we consider the relationship between any two nodes in isolation, they could either be friends (positive edge) or enemies (negative edge). Now, when we think of the group as a whole, the relationship between any two nodes would affect their relationship with the third node. For instance, if two of the users are friends with each other, they are both likely to be friends (or enemies) with the third user. In more general terms, given a set of three nodes, the relationship between the nodes is said to be *balanced* when the multiplication of all the edge signs is positive. Figure 1 presents a signed network with both balanced and unbalanced states. Green edges have a positive sign while the blue edges have a negative sign. The circles marked in yellow are examples of *balanced* states while the other two triangles (unmarked) are examples of *unbalanced* states. Based on the Structural Balance Theory, the relationship between any two nodes is likely to be consistent with the sign of the triad connecting them. If there is a (+, +) or (−, −) triad between the nodes, then it is more likely that the nodes would have a positive relation. Otherwise, a negative relation is more likely to form. Therefore, the information from the triads in a signed network can effectively complement the information from the edges for the task of inferring the relationship between the nodes. A natural application of the Structural Balance Theory, for learning node representations in a signed network, would be to use triads, along with the edges, in the given signed network.

Building on the above idea, we propose a novel framework for learning node embeddings in the context of signed networks. Our approach leverages both the Structural Balance Theory and the network structure by considering both edges and triads when learning the node embeddings. For each node, we learn two sets of node embeddings-one focusing of the sign information and the other focusing on the structure information. We combine these two embeddings based on the principle of Structural Balance Theory to predict the sign between any pair of nodes in the network. Specifically, for each pair of linked nodes, our approach samples a number of triads connecting the nodes. Then, we use the sign embeddings of nodes, along with the sampled path, to predict the sign between the given pair of nodes. Since we sample multiple triads, each of which is used to make an independent prediction, we need a mechanism to integrate the result of different predictions into a single prediction. To that end, we leverage the attention mechanism (Bahdanau et al., 2014) that enables us to assign different weights or importance to the different triads so that we can focus on the most informative triads. We describe the model and the training schemes in detail in section 4 and show that our approach can be used for both directed and undirected signed networks.

We conduct experiments on three real-world signed networks of varying sizes. Experimental results on the edge sign prediction task show that our, simple and intuitive approach, is very close in performance as compared to the state of the art approaches for learning signed network embedding.

In summary, we make the following contributions in this paper:

- We study the problem of signed network embedding, and we draw key inspirations from the Structural Balance Theory for leveraging high-order structures of a network.
- We propose a novel approach to learn node embeddings in the signed networks. Our approach leverages information from both edges and triads in the networks.
- We leverage the attention mechanism to aggregate information from different triads and edges. Our extensive experiments on 3 real-world signed networks highlight the effectiveness of our approach.

## 2. Preliminaries

### 2.1. Problem Definition

Networks are the most common choice of data structure for representing the relationship between objects. Hence networks are ubiquitous in the real world with many different forms. In this paper, we study a specific class of networks, the signed network. We formally define the signed networks to distinguish them from other class of networks.

**Definition 1**. ***(Signed Network.)*** *A **Signed Network** can be formally denoted as G = (V, E_pos_* ∪ *E_neg_). Here, V represents the set of nodes in the network. E_pos_ and E_neg_ represents the set of edges with the positive and the negative signs, respectively. These signs indicate opposite relationships*.

Signed networks are very popular in the context of social networks. For example, in the Epinions social network, users can form positive and negative edges based on the “trust” and “distrust” relations. In the Slashdot social network, users can mark other users as “friends” and “foes,” which can be treated as positive and negative edges, respectively.

In this paper, we are interested in learning node embeddings in the given signed network. The learned node embeddings are expected to preserve both the structural properties and the signed properties of the nodes in the network while being useful for various downstream tasks including the task of edge sign prediction. We now formally define our problem as follows:

**Definition 2**. ***(Signed Network Embedding)*** *Given a signed network G = (V, E_pos_* ∪ *E_neg_) and a vector dimension d* ≪ *|V|, the problem of* ***Signed Network Embedding*** *aims to learn a vector representation* {***x**_v_*} *for each node v ∈ V*.

Real-world signed networks tend to be very sparse (Table 1) and hence using just the edge information for learning the node embeddings would not be sufficient. So we propose to leverage the higher order structures in the network to learn node embeddings. Specifically, we propose to leverage the triads in the networks, which are formally defined below.

**Table 1:** Statistics of the datasets.

	**Epinions**	**Slashdot**	**Wikipedia**
No. of nodes	131,828	82,140	7,118
No. of edges	841,372	549,202	103675
% of positive edges	85.3	77.4	78.8
% of negative edges	14.7	22.6	21.2
No. of training edges	673,097	439,361	82940
% of nodes with positive degree one	38.2	25.5	24.9

**Definition 3. (*Triad*)** *Given a signed network G = (V, E_pos_* ∪ *E_neg_), a triad is a collection of 3 nodes (x, y, z) such that there exists edges between nodes (x, y), (y, z) and (x, z). If the given graph G is directed, then directed edges should exist between (x, y), (y, z), and (x, z). A triad has three signs corresponding to the three edges. We denote the sign of the triad (x, y, z) by the sign between edges (x, y) and (y, z). If the sign between the two edges are “(positive, positive),” we denote the sign of the triad to be “pos-pos.” Similarly, we use the notation “pos-neg,” “neg-pos” and “neg-neg” when edge signs are (“positive,” “negative”), (“negative,” “positive”) and (“negative,” “negative”), respectively*.

### 2.2. Structural Balance Theory

The concept of Structural Balance is grounded in the principles from social psychology (Heider, 1946) and has been extended for graphs by Harary (1953), Davis (1963), and Cartwright and Harary (1956). Consider a group of three nodes (users) where every node is related to every other node. This implies there exists an edge between any pair of nodes. The sign of each edge could either be positive (indicating friendship) or negative (indicating enmity). When the relationship between a pair of nodes is considered in isolation to the other nodes, the edge could be either positive or negative. But when the group is considered as a whole, the Structural Balance Theory puts some constraints on which configuration of signs are more likely than others.

**Figure 2:**
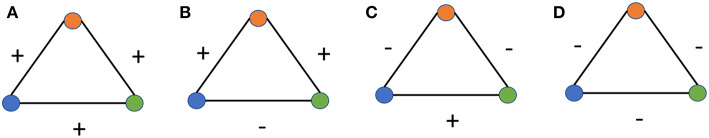
Different combinations of signs in a triad. In **(A)**, all the edges have a positive sign and the triad is *balanced*. In **(B)**, two edges have the positive sign and one edge has the negative sign. This makes the triad *unbalanced*. In **(C)**, two edges have the negative sign and one edge has the positive sign. This makes the triad *balanced*. In **(D)**, all the edges have the negative sign, which makes the triad *unbalanced*.

More specifically, it states that when the 4 distinct ways of labeling the edges are considered, as described in Figure 1, the triangles with 3 positive edges or with 2 negative edges and 1 positive edge are *balanced* (and are more likely to form) while the other two configurations are *unbalanced* (and are less likely to form). The notion of *balanced* vs. *unbalanced* states can be explained in terms of the interpersonal relationships between the nodes. In Figure 2A, the 3 nodes are mutual friends and in Figure 2C, two nodes are friends with each other and have a mutual enemy. These 2 kinds of relationships are natural and more likely. In Figure 2B, the orange node is friends with both green and blue nodes but they themselves are not friends. In such a scenario, the orange node would either try to bring green and blue nodes closer and change the negative sign of the edge between them to positive or would side with either one of them, hence turning one of its positive edges to negative. Similarly, in Figure 2D, all the 3 nodes are enemies with each other and two of them are likely to join forces against the third (based on the conventional wisdom that *enemy of my enemy is my friend*) thereby turning one of the negative edges to positive. In both Figures 2B,D there is some kind of social unbalance or strain that could be remedied by having an odd number of positive edges. This is the crux of Structural Balance Theory-in signed networks, when groups of 3 completely connected nodes (triads or cliques of size 3) are considered, the configurations with an odd number of positive edges are more likely to occur than configurations with an even number of positive edges. This insight provides the inspiration for our proposed approach where we leverage the information about paths in the network when predicting the sign between the edges.

Structural Balance Theory is one of the reasons why the approaches developed for unsigned networks cannot be directly applied for signed networks. In unsigned networks, a node is more likely to be similar to its second order neighbors as compared to a node with which it is not connected even in terms of second order neighbors. In signed networks, the nature of the relationship between second-order nodes depends on the nature of the path connecting them. A node is more likely to have a positive edge with “friends of their friends” and a negative edge with “enemy of their friends.” Such dependence is not exhibited by the unsigned networks.

## 3. Related Work

We propose to leverage the Structural Balance Theory along with the network structure to learn node representations for a given signed network. We further use the attention mechanism to aggregate the predictions from the different higher order structures. As such, our work is related to the paradigm of node representation learning, to the Structural Balance Theory and to the attention mechanism.

### 3.1. Node Representation Learning

Given a network, the goal of node representation learning techniques is to embed the network into a low-dimensional vector space, where every node is represented as a dense, real-valued vector. The learned node representations are expected to preserve the properties of the nodes and have been shown to perform well across various tasks, including node classification (Perozzi et al., 2014), link prediction (Grover and Leskovec, 2016) and network visualization (Tang et al., 2016). Most node representation learning algorithms (Perozzi et al., 2014; Tang et al., 2015) focus on unsigned networks, in which only a single type of edge exists. A different class of approaches focuses on using matrix factorization based techniques to embed the high dimensional adjacency matrix into a low dimensional vector space. Techniques like eigendecomposition of the Laplacian matrix (Chung, 1997) and factorizing the k-step transition matrix (Cao et al., 2015) fall under this category. Different from these studies, in this paper we focus on the task of learning representation for the signed networks, where the edges have two opposite signs and represent contradictory relationships.

There are also some studies working on node representation learning in signed networks (Wang et al., 2017a,b; Wang H. et al., 2017). Typically, these methods will learn a classifier to discriminate between the positive, the negative and the unlabeled edges. The idea behind these approaches is that the learned node representations can well preserve both the positive and the negative relations in the network which is helpful in achieving impressive results on link prediction task (Wang et al., 2017b). However, these methods only consider the local structure of the network. More specifically, they consider only the observed edges (i.e., positive edges and negative edges) for training the model. In many real-life networks, the observed edges can be very scarce and these approaches suffer from the problem of data sparsity. Our approach circumvents the problem by considering the high-order structure of the given network. Specifically, we draw inspiration from the Structural Balance Theory and leverage the triads in the network when learning the node representations.

Guha et al. (2004) was one of the earliest works that proposed to model the problem of predicting edge signs in a social network as a computational problem. They proposed a framework based on the trust propagation schemes based on the idea that a user is much more likely to believe statements from a trusted friend than from a stranger. The framework could be used to build a *web of trust* and predict trust and distrust between a given pair of users. Given the adjacency matrix corresponding to the signed network, a *combined* matrix is computed. This *combined* matrix is equivalent to the one-step propagation of trust in the signed network. The linear combinations of the higher powers of this *combined matrix* are computed to propagate the trust and distrust effects further in the network. Unlike our approach, Guha et al. (2004) does not model the problem as that of representation learning. Further, it does not seek to leverage theories grounded in social sciences.

### 3.2. Structural Balance Theory

Leskovec et al. (2010) was one of the earliest works to explore the *edge sign prediction problem* under the light of social-psychological theories of balance and statues (Heider, 1946; Cartwright and Harary, 1956). The underlying idea is that the sign of the edge *(a, b)* should be such that it minimizes the number of unbalanced triangles which have *(a, b)* as one of its edges. In the context of the Structural Balance Theory, an unbalanced triangle is defined as a triangle with an odd number of negative edges. If we denote the positive edges as “+1” and negative edges as “-1,” the product of the edges, in a balanced triangle, is positive while it is negative in an unbalanced triangle. Leskovec et al. (2010) employ supervised learning approaches and use two class of features. The first class of features is based on (signed) degree of nodes and includes the number of incoming (and outgoing) positive (as well as negative) edges. The second class of features is based on the principles from social-psychological theories and uses the count of the different kind of triads (or triangles) in the given network. The paper reported results on 3 datasets and just like (Guha et al., 2004), adopted a leave-one-out cross-validation approach. While this approach works very well in practice, it relies on hand engineering the features, In contrast, we model the problem from the perspective of representation learning and do not need hand-crafted features.

Chiang et al. (2011) extends the idea of leveraging features guided by theories from social sciences and describe many measures of imbalance that can be derived from Social Balance Theory and Signed Graph Theory. Further, they show that the performance of the model can be improved if the measure of imbalance depends on *higher order cycles* in the network. This effect is more predominant for sparse graphs where the edges are scarce. Like their approach, our model also uses higher order structures in the network but the emphasis of our model is on learning the representation of the network. Further our approach does not need different handcrafted measures of social imbalance.

Recently, Kim et al. (2018) proposed a general network embedding method that can represent both the sign and the direction of edges in the embedding space. They formulate (and optimize) likelihood functions over both the direct and indirect signed networks and their approach outperforms all the other approaches we have discussed so far. Hence we consider their model among the baselines that we use to evaluate our proposed model. Just like their approach, our approach works for both directed and undirected signed networks (explained in detail in section 4.7). Our work is different from their work as we leverage both triads (based on principles from Structural Balance Theory) and attention mechanism (to assign an importance weight to predictions corresponding to different triads).

### 3.3. Attention Mechanism

When predicting the sign for an edge *(a, b)*, our approach sample multiple higher order structures which connect the nodes *a* and *b*. For each of the sampled paths, the model makes a prediction for the sign of the edge *(a, b)*. Sampling multiple higher order structures lead to multiple predictions which need to be aggregated into one prediction. For aggregating these predictions, we use the attention mechanism where the model attends to each higher order structure and assigns a weight to the prediction corresponding to that structure. Attention mechanism was first proposed in the context of machine translation systems by Bahdanau et al. (2014). Since then, it has been successfully applies to many applications including image classification (Mnih et al., 2014), question answering (Seo et al., 2016) and graph representation learning (Lin et al., 2016) among others (Vaswani et al., 2017). While our proposed method draws inspirations from the prior work on attention, we apply the idea of using attention to a new application ie learning representation for a signed network. This has not been explored before to the best of our knowledge.

## 4. Model

In this section, we introduce our approach for learning representations for signed networks. Our proposed model can be described in terms of three broad ideas. The first idea is that in the case of signed networks, each node has two kinds of attributes (or properties)-one which affects which other nodes would it link to and the other which affects the sign of those links. Given these two related but different tasks, we propose to have two set of embeddings which are jointly used for predicting the sign of a given edge. The second idea is that the Structural Balance Theory provides us with a way to leverage the higher order structures along with the edges for learning the network representation. The third idea is that attention mechanism can provide an effective technique for combining predictions made using multiple higher order structures. Note that we will first describe all the three ideas assuming the network to be undirected. Then we would describe how the proposed approach can be easily extended for the case of directed networks (section 4.7). In fact, all the network datasets that we consider for evaluating our proposed model are directed in nature which shows that our approach works quite well on the directed networks.

We now describe each of the three ideas in detail.

### 4.1. Two Sets of Node Embedding

In the case of an unsigned network, the network structure can be described just in terms of edge connections (which gives the degree of proximity or closeness between the nodes) while in case of signed networks, the network structure needs to be described in terms of both edge connections and the sign of the edges. Hence, we propose to learn two sets of embeddings for each node. The first set of node embeddings is referred to as the *structural* node embeddings and are denoted by *Emb*_*struct*_. Here the subscript “struct” indicates that these embeddings are learned while focusing only on the structural (or edge) connectivity. The sign of the edges is ignored while training *Emb*_*struct*_. Since this learning phase treats the network to be an unsigned network, we can use any of the existing unsigned node embedding techniques and in particular, we use the LINE embedding technique as described in Tang et al. (2015). LINE uses an edge sampling algorithm to tune a carefully designed objective function that can preserve both the local and global network structures. We used LINE given that it is shown to work with many different classes of networks: undirected, directed, and/or weighted. Note that we plug in any other node embedding learning technique here (like Perozzi et al., 2014; Grover and Leskovec, 2016) as the downstream model is independent of the node embedding technique used here.

As we have already discussed (section 2.2), treating the signed network as an unsigned network is not a sound strategy and thus we learn another set of node embeddings which we refer to as the *signed* node embedding and denote as *Emb*_*sign*_. Here the subscript “sign” indicates that these embeddings are learned with the objective of predicting the correct sign between a given pair of nodes (*a, b*). The *Emb*_*sign*_ embeddings are be trained in the following manner: Consider the edge between two nodes *a* and *b*. If the edge sign is positive, we denote the “true sign” as 1 otherwise as 0. We compute the probability of the sign, between node *a* and *b* to be positive, as

(1) $$\begin{array}{r} p_{sign}^{edge}\left(a,b\right)=\sigma \left(Emb_{sign}\left(a\right).Emb_{sign}{\left(b\right)}^{T}\right) \end{array}$$

$p_{sign}^{edge}\left(a,b\right)$ denotes the probability of the edge sign, between nodes *a* and *b*, as predicted by the *sign* embedding model. Here the superscript “edge” indicates that the prediction is made using the nodes that make up the edge. Then we minimize the cross-entropy loss between the predicted sign and the actual sign. This loss is used to train the *Emb*_*sign*_ embeddings. We discuss some optimizations in the training procedure in section 4.5.

### 4.2. Leveraging Higher Order Structures Using Structural Balance Theory

Most existing network embedding techniques only consider the edges (i.e., positive and negative edges) in the given network when learning network representation. When the edges in networks are scarce, their performance can be very limited. Our approach addresses this challenge by considering the high-order structures such as triads (definition 3) in the network. We draw inspiration from the Structural Balance Theory (Cartwright and Harary, 1956), which states that nodes would prefer forming signed edges such that a balanced state emerges in their interpersonal relationships. The theory implies that the sign between a pair of nodes should be consistent with the sign of triads containing them. This motivates us to leverage the triad as a feature for the task of Signed Network Embedding. Note that we consider only triads as it is very efficient to enumerate all possible triads in a given network (Azad et al., 2015). In general, our approach can be easily extended for other higher level structures as well.

We now describe how we model triads for predicting the edge sign.

### 4.3. Modeling Each Triad

Consider a triad (definition 3) of the form [*a, b, c*] which comprises of the edges (*a, b*), (*b, c*), and (*a, c*). We want to predict the sign between nodes *a* and *c*. If the signs for the edges (*a*.*b*) and (*b*.*c*) are known beforehand, we can compute the *sign*(*a, c*) based on the Structural Balance Theory as follows:

(2) $$\begin{array}{r} sign\left(a,c\right)=xnor\left(sign\left(a,b\right),sign\left(b,c\right)\right) \end{array}$$

Here *xnor* refers to the Exclusive NOR operation and is also known as *enor*, *exnor* or *nxor*.

There are two downsides to directly using this approach (Equation 2). The first downside is that in the real-life setting, we do not know the sign for all the edges, So even if we know that there is an edge between *a* and *b* and between *b* and *c*, we may not know the nature of the relationship between them (ie the sign of edge between them). The second downside is that if we only rely on edges where the sign is known beforehand, we can not apply any learning technique as our “features” (in this case, *sign*(*a, b*) and *sign*(*b, c*)) are already fixed.

We get around both of these problems by using the “sign” embeddings *Emb*_*sign*_ which are trained to predict the sign for a given edge ie we use the *Emb*_*sign*_ embeddings to predict the sign for edge (*a, b*) and (*b, c*). Let the predicted signs be denoted as $p_{sign}^{edge}\left(a,b\right)$ and $p_{sign}^{edge}\left(b,c\right)$ respectively. Now the *xnor* operation is described for discrete, boolean symbols while our probabilities are real numbers. We have two options here: First option is that we map the probabilities to the predicted sign (which is 0 or 1). Since this would introduce a non-differentiable component in the pipeline, we would have to use REINFORCE-style algorithm (Williams, 1992) for training the model.

Alternatively, we could approximate the *xnor* operator for two real numbers *α* and *β* (which are in the range [0, 1]) as follows:

(3) $$\begin{array}{r} xnor\left(\alpha ,\beta \right)=\alpha *\beta +\left(1-\alpha \right)*\left(1-\beta \right) \end{array}$$

This is analogous to the logical form of *xnor* (between two boolean variables *a* and *b*) which can be written as:

(4) $$\begin{array}{r} xnor\left(a,b\right)=\left(a\;and\;b\right)\;or\;\left(not\;a\;and\;not\;b\right) \end{array}$$

Assuming that both $p_{sign}^{edge}\left(a,b\right)$ and $p_{sign}^{edge}\left(b,c\right)$ are real numbers in the range [0, 1],(which we ensured by normalizing the values so that we could interpret these numbers as the probability of the edge being positive) we could predict the sign for edge between *a* and *b* as $p_{sign}^{triad}\left(a,c\right)$ as follows:

(5) $$\begin{array}{r} p_{sign}^{triad}(a,c)=p_{sign}^{edge}(a,b)*p_{sign}^{edge}(b,c)+(1-p_{sign}^{edge}(a,b))*(1-p_{sign}^{edge}(b,c)) \end{array}$$

Here the superscript “triad” indicates that the prediction is made using all the nodes in the triad while the superscript “edge” indicates that the prediction is made using only the nodes that make up the edge.

Given the true value of *sign*(*a, c*), we can minimize the cross-entropy loss between the true value and the predicted value of the sign. The resulting loss would be used to update the node embeddings of all the three nodes *a*, *b* and *c*.

We describe how our approach is different from the other works like (Kim et al., 2018) which also propose to use the Structural Balance Theory for predicting signs of higher order structures. In those cases, the idea is to sample a walk of nodes (*a, b, c, d*…) where the sign between every consecutive pair of nodes ie (*a, b*), (*b, c*) etc are known. The Structural Balance Theory is used to annotate extra pairs of nodes ie (*a, c*), (*a, d*) etc and then use them as part of the training data. Hence, in those approaches, the sign for all the edges comprising of the walk should be known. In our case, we do not need to know the sign of the triad as we can infer the sign using the “sign” embedding model *Emb*_*sign*_. Further, the Structural Balance Theory describes a social phenomenon and is not an absolute physical law. Thus, it can make incorrect predictions. So when we use the theory to augment the dataset by annotating unseen edges, it would lead to incorrect training data being mixed with the correct data. The idea there is to account for Structural Balance Theory by augmenting the dataset. In our case, we never modify the given true data and include the effect of the Structural Balance Theory by augmenting the prediction mechanism. This allows the model the encode the knowledge of Structural Balance Theory without having to train on spurious examples. In the next section, we describe how we make our model even more robust by sampling multiple triads, computing predictions corresponding to each triad and aggregating the predictions by means of attention mechanism.

### 4.4. Integrating Multiple Triads Using Attention

So far, we have considered a node embedding model for learning representation for a given signed network. Then we discussed how *Emb*_*sign*_ model can be extended to incorporate the effect of Structural Balance Theory by considering higher order structures, namely triads, in the network. In this section, we explore the third idea that informs the design of our model.

The third idea is to sample multiple triads (and not just one) when we want to predict the sign for a given edge (*a, c*) as this helps to leverage even more information from the network. Given the edge (*a, c*), we sample *n* triads where the *i*^*th*^ triad is represented as (*a, b*^*i*^, *c*). For each of the *n* triads, we make independent predictions as described in Equation 5. That is, for the *i*^*th*^ triad, we have the predicted sign $p_{sign}^{i}\left(a,c\right)$ given as:

(6) $$\begin{array}{r} p_{sign}^{i}(a,c)=p_{sign}^{edge}(a,b^{i})p_{sign}^{edge}(b^{i},c)+(1-p_{sign}^{edge}(a,b^{i}))*(1-p_{sign}^{edge}(b^{i},c)) \end{array}$$

We can also predict the sign between nodes *a* and *c* using the *sign* embedding model *Emb*_*sign*_ as well. We denote the prediction obtained in this way as $p_{sign}^{edge}\left(a,c\right)$.

Now, we have *n* + 1 predictions corresponding to the sign for the edge (*a, c*) and we need to aggregate these predictions into a single prediction. A straight-forward approach is to combine by these predictions by performing simple averaging. This approach is not likely to be very effective as the different features (different triads and edges) are not likely to be equally informative. Hence we use the attention mechanism to assign weights to each prediction and then perform a weighted averaging over the different predictions.

To obtain the attention weights, we need some notion of similarity which can guide the model to decide how much weight should be placed on each prediction. Recall that we have *n* tuples of the form (*a, b*^*i*^, *c*) and an edge of the form (*a, c*). For computing the attention scores, we use the *structural* embedding, represented as *Emb*_*struct*_. The key benefit here is that the *structural* embeddings have been trained to capture node similarity (irrespective of the sign). Using *Emb*_*struct*_ allows the model to assign attention weights independently to each triad, irrespective of its prediction.

Formally, we encode the edge (*a, b*) into a vector by adding the *structural* embeddings corresponding to node *a* and *b* as shown:

(7) $$\begin{array}{r} E_{edge}\left(a,c\right)=\frac{Emb_{struct}\left(a\right)+Emb_{struct}\left(c\right)}{2} \end{array}$$

Similarly, the triad (*a, b*^*i*^, *c*) is also encoded into a vector by adding the structural embeddings correspponding to nodes *a*, *b*^*i*^ and *c* as shown:

(8) $$\begin{array}{r} E_{triad}\left(a,b^{i},c\right)=\frac{Emb_{struct}\left(a\right)+Emb_{struct}\left(b^{i}\right)+Emb_{struct}\left(c\right)}{3} \end{array}$$

**Figure 3:**
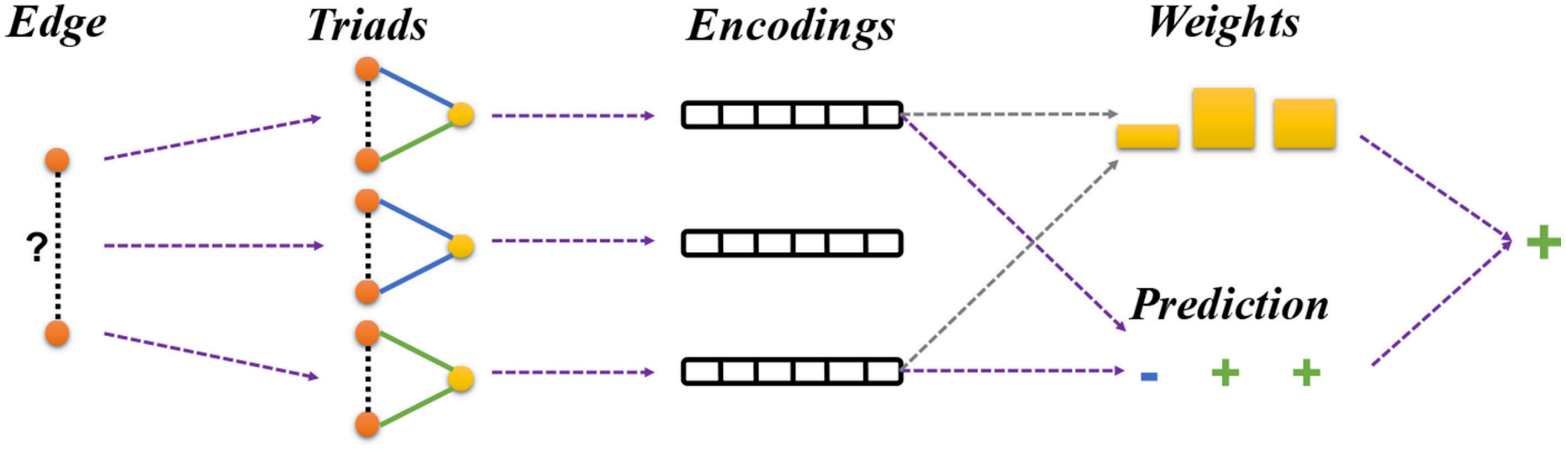
Framework overview. Given a signed network, our framework learns node embeddings by predicting the sign of each training edge. For each pair of the linked nodes, we first sample a batch of triads that connect the given nodes. The triads are used to make the prediction about the sign of the given edge as described in section 4.3 (Equation 5). Finally, the prediction results from different triads are integrated using an attention mechanism as described in section 4.4 (Equation 10).

We can then compute the similarity score corresponding to each triad (*a, b*^*i*^, *c*) as the dot-product between the representation of *edge*(*a, c*) (given by *E*_*edge*_(*a, c*)) and representation of *triad*(*a, b*^*i*^, *c*) (given by $E_{triad}\left(a,b^{i},c\right)$). This is similar to the dot-product attention used in Luong et al. (2015). The similarity score assigned to the prediction $p_{sign}^{edge}\left(a,c\right)$ is 1. The similarity scores are then normalized by dividing by the sum of all the similarity scores to obtain the attention scores. For the *i*^*th*^ triad, the attention score λ^*i*^ is given as :

(9) $$\begin{array}{r} \lambda_{i}=\frac{\exp\left(E_{edge}{\left(a,c\right)}^{T}E_{triad}\left(a,b^{i},c\right)\right)}{Z} \end{array}$$

where *Z* is the normalization constant. Basically, If the triad embedding has a large bilinear product with the edge embedding, it means that the nodes *a* and *b* believe the triad is informative and the triad would have a large weight in the final prediction. These steps have been illustrated in Figure 3.

Formally, for a pair of nodes *a* and *c*, we will sample a mini-batch of triads consisting of the two given nodes. The size of the mini-batch *n* is an hyper-parameter. If there are less than *n* triads between *a* and *c*, we will pad the batch with some dummy triads [*a*, < *pad* >, *c*] that we filter out during training. Each of the *k* triads is used to make a prediction about the edge (*a, c*) using the Equation 1. Then we use the *sign* embedding corresponding to nodes *a* and *b* to make the prediction about the sign of the given edge using Equation 5. Attention scores λ_*i*_ are computed for each of the predictions as given by Equation 9.

Once we have obtained the attention scores over different triads, we perform a weighted averaging over the predictions of the different triads, using the attention scores as the weights. We present the formal equation:

(10) $$\begin{array}{r} p_{sign}\left(a,c\right)=\lambda_{0}*p_{sign}^{edge}\left(a,c\right)+\overset{n}{\underset{i=1}{\sum }}\lambda_{i}*p_{sign}^{triad}\left(a,b^{i},c\right) \end{array}$$

During training, we expect the predicted sign between node *a* and *c* could be consistent with the real sign. Therefore, the cross-entropy between the predicted sign and the real sign is treated as the objective function.

### 4.5. Optimization

The only trainable components in our model are the embeddings - *Emb*_*struct*_ and *Emb*_*sign*_. The entire framework can be effectively optimized, end-to-end, through the backpropagation algorithm. We summarize the different steps in the process in Algorithm 1.

**Algorithm 1:** Learning Signed Network Embedding.

**Require:** Signed network *G*
**Ensure:** Learned Node embeddings
1: Train the structural node embedding using LINE (section 4.1)
2: **while** not converge **do**
3: Sample an edge (*a, c*) from *G*.
4: Predict $p_{sign}^{edge}\left(a,c\right)$ (Equation 1).
5: Compute edge encoding (Equation 7).
6: Sample *n* triads ${\left\{\left(a,b^{i},c\right)\right\}}_{i=1}^{n}$ which connects *a* and *c*.
7: **for** each triad (*a, b*^*i*^, *c*) **do**
8: Predict $p_{sign}^{triad}\left(a,c\right)$ (Equation 5).
9: Compute triad encoding (Equation 8).
10: **end for**
11: Calculate the attention over different triads (Equation 9).
12: Calculate the final prediction (Equation 10).
13: Compare the predicted sign of (*a, c*) with the real sign.
14: Perform backpropagation to update parameters.
15: **end while**

We now describe some strategies that we used to accelerate the training.

The *Structural* embeddings are only used during when assigning attention scores to different triads. Hence, we decided to pre-train the node embeddings using LINE (Tang et al., 2015) and only slowly finetune them when computing attention scores. This has many benefits:

1. Since the *Emb*_*struct*_ model is trained independently, we are free to use any technique for learning the embeddings irrespective of the other components. For example, we could use a random walk based approach even though the other components in the pipeline do not use random walks for training.
2. Since the embeddings are pretrained, the results from the attention module are quite good right from the start of the training. This makes it easier to tune the other components.
3. We could re-use the pretrained embedding model across different runs which reduces the overall time to train and validate the models.

Inspired by the benefit of using pretrained *Emb*_*struct*_ embeddings, we perform pretraining for the *Emb*_*sign*_ embeddings as well where we do not consider any triads (and hence any attention weights). This pretrained model is further trained via sampling triads and the pretraining is seen as a means to provide a good initialization to the model.

We note that we tried training the model without the pretraining steps as well. It takes longer time (due to the required fine-tuning of many more hyper-parameters) when all the components are being trained simultaneously from scratch. Hence the main contribution of these optimization tricks is to accelerate the end-to-end workflow.

Table 1 highlights the high-class imbalance between the positive and the negative edges. While learning the *sign* embedding, we account for this imbalance by sampling the negative edges with approx 2x frequency as compared to the positive edges. Oversampling negative edges seems to be crucial for learning useful network representation.

Another optimization we did was to separately handle the nodes with a degree of 1. As shown in Table 1, around 25–30% of the nodes in the network have a positive degree of one. It is quite difficult to learn good representations for these nodes (as they have only 1 neighbor) and they slow down the training procedure. Some works like Wang et al. (2017b) remove such nodes from the training data. Instead, we follow the scheme proposed by Kim et al. (2018) where the nodes with positive degree-one are eliminated and the embedding is learned for the rest of the network. Then each of the eliminated nodes is assigned the embedding corresponding to its only neighbor ie the eliminated nodes share the embedding with their only neighbor.

### 4.6. Inference

Once the training process converges, the learned node embeddings can be leveraged for various downstream tasks. One popular example is to predict the sign of a potential edge (*a, c*). For this task, we first sample a batch of triads connecting both the nodes. For each triad, we make a prediction using Equation 5. Further, we use just the nodes *a* and *c* to make the prediction for their sign (i.e., $P_{sign}^{edge}\left(a,b\right)$). Then we compute the attention scores using equation 9 and aggregate the different predictions to obtain the final prediction.

Consider a sampled triad (*a, b*^*i*^, *c*) where the task is to predict the sign of the edge (*a, c*). To predict $p_{sign}^{triad}\left(a,c\right)$, we could either use the actual signs for edges (*a, b*^*i*^) and (*b*^*i*^, *c*) or could use the predicted signs ($p_{sign}^{edge}\left(a,b^{i}\right)$ and $p_{sign}^{edge}\left(b^{i},c\right)$). In section 4.3, we describe the rationale behind choosing the second approach during training. But during evaluation, we are free to use either approach. In practice, we tried three variants-always use true signs, always use predicted signs, and mix both true and predicted signs. We find that always using predicted signs or mixing both true and predicted signs works better than always using just the true signs. In case of predicted signs, the predictions are real values between 0 and 1 and is not a hard 0/1 prediction which probably gives some more flexibility to the model (in terms of expressing confidence in an edge being positive or negative).

### 4.7. Directed Graph

So far, we have described our proposed approach assuming that the given signed network is undirected in nature. Now we discuss how our model can be applied in the context of the directed network. Note that all the datasets that we consider for evaluation are in fact directed signed networks (section 5.1). The only learnable components in our model are the two embedding layers: *Emb*_*struct*_ and *Emb*_*sign*_. A very straightforward way to extend them for directed networks is to consider separate embeddings for the nodes depending on whether they are the incoming node for an edge or the outgoing node. We denote the incoming *structural* and *signed* node embeddings as $Emb_{struct}^{in}$ and $Emb_{sign}^{in}$, respectively. Similarly, we denote the outgoing *structural* and *signed* node embeddings as $Emb_{struct}^{out}$ and $Emb_{sign}^{out}$ respectively. Now we can re-express the main equations from previous sections for the case of directed signed networks. Note that a directed edge (*a, b*) means that the *a* in the outgoing node and *b* is the incoming node.

Equation 1, which predicts the sign of an edge as function of its nodes can be written as:

(11) $$\begin{array}{r} p_{sign}^{edge}\left(a,b\right)=\sigma \left(Emb_{sign}^{out}\left(a\right).Emb_{sign}^{in}{\left(b\right)}^{T}\right) \end{array}$$

Equation 7, which encodes an edge into a continuous vector can be written as:

(12) $$\begin{array}{r} E_{edge}\left(a,c\right)=\frac{Emb_{struct}^{out}\left(a\right)+Emb_{struct}^{in}\left(c\right)}{2} \end{array}$$

Equation 8, which encodes a triad into a continuous vector can be written as:

(13) $$\begin{array}{l} E_{triad}\left(a,b^{i},c\right)\\ =\frac{Emb_{struct}^{out}\left(a\right)+Emb_{struct}^{in}\left(b^{i}\right)+Emb_{struct}^{out}\left(b^{i}\right)+Emb_{struct}^{in}\left(c\right)}{4} \end{array}$$

## 5. Experiment

Our empirical protocol ensures a rigorous evaluation of the proposed framework on several real-world signed network datasets. We compare against a wide variety of models-models that use handcrafted features, models that were originally proposed for unsigned networks, models using matrix factorization based approaches and deep learning based models that are specifically designed for the task for learning signed network embedding. We show that despite being very intuitive and highly modular, our proposed approach performs very well against these different classes of models.

We now introduce the datasets, baselines models and the experimental setup.

### 5.1. Datasets

We evaluated our proposed approach on three real-world signed networks datasets. Although all these networks are directed in nature, our model does not make any assumption about the input graph being directed and can work with both directed and undirected graphs as discussed in section 4.7.

The summary-statistics for all the three signed network is available in Table 1. Further, the frequency of different triad types (in term of signs) is described in Table 2. The datasets are publicly available ^3^. We evaluate our dataset on graphs having both a large number of nodes (>100 K) and very few nodes (<10 k).

1. **Epinions** is a social network that captures the “who-trust-whom” relationship between the users of the consumer review site epinions.com. The members can mark which community members they want to “trust” (thereby making positive links) or “distrust” (thereby negative links). this network of the trust-based relationship becomes a signed network.
2. **SlashDot** is a technology-related news website where members can tag other members as “friends” (positive links) or “foes” (negative links) thereby creating explicit positive/negative relationships within the social community.
3. **Wikipedia** is a community authored encyclopedia. Wikipedia hosts elections to determine which users should be promoted to the admin role. A user could vote in favor of a candidate (thus making a positive edge) or vote against a candidate (thus making a negative edge) or abstain from voting (this leads to absent edge). For our work, we focus only on the signed network formed by the positive and negative edges and do not consider the case of abstained voting.

**Table 2:** Triad statistics of the datasets.

	**Epinions**	**Slahsdot**	**Wikipedia**
% of + + + triads	87	84	70.2
% of + - - triads	7.1	7.2	20.7
% of + + - triads	5.2	7.7	8
% of - - - triads	0.7	1.1	11

There are some common observations which can be made in reference to all the datasets: First, all the datasets are highly unbalanced, not just in terms of frequency of positive vs. negative edges (around 80% positive edges as shown in Table 1) but also in terms of frequency of different type of triads (over 70% of the triads are of type “+ + +” as shown in Table 2). Secondly, the percentage of nodes with positive degree one is quite high (up to 38%) shown in Table 1. This highlights the need for treating these nodes separately in order to save on redundant computations (section 4.5).

### 5.2. Experiment Setup

In this section, we describe the general experimental setup that we used. We describe how the dataset splits were created, what metrics were used (and why) and include other implementation specific details that would be helpful for others.

#### 5.2.1. Dataset Splits

The data sets do not have a standard train-val-test split and different works use different splits. Following Kim et al. (2018), we perform a 5 fold cross-validation approach. During the evaluation stage, the learned node representations are used to predict the sign of edges that were not seen during training. Since our model uses information about the triads (triangles) in the given network, we took extra care to ensure that while enumerating the triads, we consider only those edges which are “visible” in the training data. This ensures that none of the “test” edges are seen by the model even as part of the sampled triad.

#### 5.2.2. Evaluation Metrics

Given the input signed network, the downstream task is to predict the sign for any edge in the network. Since, for a given edge, edge-sign can either be *positive* or *negative*, we formulate the problem as a two-class classification problem. A common characteristic of the real-life signed networks is that they are highly biased toward positive edges (as seen in Table 1). In the datasets that we consider, the ratio of the number of positive and negative is roughly 4:1. this high-class imbalance makes the Micro-F1 (accuracy) score a misleading metric for performance and we report the AUC metric along with the Micro-F1 score. AUC is more robust to class imbalance.

#### 5.2.3. Implementation Details

We have implemented our proposed models using PyTorch (version 0.3.1) from Paszke et al. (2017). Binary cross-entropy loss and Adam optimizer (Kingma and Ba, 2014), are used for all the experiments. Early stopping is used to prevent overfitting. The size of both the “structural” embedding and the “sign” embedding are kept fixed at 100 and the number of triads sampled is fixed at 5. We tried some other embedding dimensions and number of triads and found the results to be quite stable across these parameters. When using the LINE model, we follow all the recommendations as specified in the paper (Tang et al., 2015). When pretraining the “structural” embedding and the “sign” embedding, we use a part of the training data as the validation data to decide when to stop training. For the baselines, we use the results reported in Kim et al. (2018) as they do a thorough evaluation with 6 baselines. The largest model is trained with 8 processes and takes around 30 min to converge (though in practice we ran it longer to ensure that it had actually converged).

### 5.3. Baselines

We refer to our proposed model as the **TEA** (**T**riad+**E**dge+**A**ttention) model. This model uses both triad features and edge features (in accordance with the Structure Balance Theory) for learning the representation for the different nodes in the given signed network. It further uses the attention mechanism to compute a weighted aggregate of the predictions corresponding to the edge and the different triads. We compare the **TEA** model with following models and variants:

1. **Count-Based (CB) Model**: Leskovec et al. (2010) proposed the use of two class of count-based features - features based on the (signed) degree of nodes and the features based on the count for different kind of triads.
2. **Node2Vec (N2V) Model**: Grover and Leskovec (2016) proposed a method to learn node embedding for unsigned networks by performing random walks on the network. The method is modified for the signed network by treating only the positive edges as actual edges and ignoring the negative edges.
3. **Matrix Factorization (MF) Model**: Hsieh et al. (2012) proposed to perform matrix factorization to learn a low rank representation of the given signed network.
4. **Balanced Normalized Signed Laplacian (BNS) model**: Zheng and Skillicorn (2015) proposed two spectral approaches for modeling and analyzing the signed graphs based on the random walk normalized Laplacian matrix.
5. **Signed Network Embedding (SNE) Model**: Yuan et al. (2017) proposed to use the log-bilinear model to learn node representation for all the nodes sampled along a random walk. in conjunction with the random walks. It also incorporates two signed-type vectors to capture the positive or negative relationship of each edge along the path.
6. **Signed Network Embedding (SiNE) Model**: Wang et al. (2017b) proposed to learn node embeddings by discriminating the positive edges from the negative and unlabeled edges.
7. **SIDE: Representation Learning in Signed Directed Networks**: Kim et al. (2018) proposed a new network embedding method that can represent both the sign and the direction of edges in the embedding space by use of a carefully formulated likelihood objective.
8. **TE Model**: A variant of the **TEA** model which considers multiple triads in the signed network but does not use the attention mechanism. Instead, the predictions corresponding to the different triads are given the same weight when aggregating the predictions corresponding to the different triads.

We note that for the baselines, we are reporting results from the work done by Kim et al. (2018) as they did a thorough evaluation with 6 baselines. We ensure that we are using the same dataset as them and are performing a 5 fold cross validation just liked they did to make sure our results are comparable to theirs. Additionally, we re-implemented the CB model and can confirm that our results are quite close to the results reported in the Leskovec et al. (2010) paper. We discuss these results in detail in section 5.4.

### 5.4. Results

In this section, we compare the performance of our proposed **TEA** model with the baselines and the variants (described in section 5.3) on the different datasets (described in section 5.1). The results have been summarized in Table 3.

**Table 3:** Quantitative results in the link prediction task.

**Model**	**Epinions**		**Slashdot**		**Wiki**	
	**AUC**	**Micro-F1**	**AUC**	**Micro-F1**	**AUC**	**Micro-F1**
CB	0.951	0.960	0.889	0.906	0.879	0.907
N2V	0.764	0.893	0.697	0.811	0.648	0.879
MF	0.920	0.957	0.877	0.910	0.875	0.913
BNS	0.893	0.948	0.842	0.895	0.861	0.901
SNE	0.820	0.924	0.746	0.874	0.762	0.882
SiNE	0.860	0.922	0.816	0.887	0.790	0.882
SIDE	0.967	0.972	0.889	0.911	0.901	0.918
TE	0.923	0.943	0.851	0.872	0.875	0.891
**TEA**	0.959	0.966	0.878	0.898	0.90	0.92

The Count-Based (CB) model uses simple features like node degree and frequency of different kind of triads. While the model is quite straightforward and intuitive, it actually performs quite well as compared to the more sophisticated baselines. One reason could be that the **CB** model uses the hand-picked features which are very well suited for the task of predicting the edge sign. The poor performance of **N2V** is not surprising as the technique is designed for unsigned networks and it is unfair to compare it with techniques that are designed specifically for signed networks. An important trend that applies to most of the approaches considered here is the wide gap in their performance in terms of AUC and the F1 score. This suggests that the models are able to pick on the data imbalance which helps on the metrics like F1-score but not on metrics like AUC which are more robust to data imbalance.

We observe that our proposed **TEA** model consistently performs better than most of the baselines and is quite close in performance to the best performing Representation Learning in Signed Directed Networks **(SIDE)** model. This is despite the fact that the **TEA** model is very simple (the only trainable parameters are the node embeddings) and highly modular. Specifically, our model decouples the modules for learning *structural* embedding, *signed* embedding and *triad* embedding. For example, we could use the **SIDE** model in place of our *Emb*_*sign*_ module and finetune over the triad data using the attention mechanism. Also note that both **SIDE** and TEA models leverage both the higher order structures and the Structural Balance Theory, albeit in different ways (as described in section 3. For instance, we incorporate the effect of the Structural Balance Theory by using triads for sign prediction while others use the theory of augmenting the dataset. Further, the use of attention mechanism is unique to our model and is not used by other models. In the next section, we study the effect of attention mechanism by means of some ablation studies (section 5.5).

### 5.5. Ablation Study

In this section, we study how the use of attention mechanism contribute to the performance of the complete model. To that end, we consider a variant of the **TEA** model which does not use attention mechanism. The resulting model is referred to as the **TE** model (Table 3). This model still sample multiple triads but then performs an unweighted averaging of the prediction for the different triads. We observe that without the use of the attention mechanism, the model's performance falls consistently by 2–3% across all the datasets. We suspect that given the high imbalance ratio between triads of different signs (Table 2), the model is more likely to sample the “pos-pos” triads as compared to any other kind of triads. In the absence of the attention mechanism, all the triads are given equal weight. This biases the model to be affectd more by the “pos-pos” triads and this results in the fall in the performance for both the metrics. With the attention mechanism, the model can choose which triads are important for a given edge and reduce the importance of other triads there by overcoming the sample bias. In section 5.6, we present some actual examples of this kind of biases from the datasets.

We consider one more abalation study where we neither consider the attention mechanism nor do we use the triads. In that case, the model is just learning embeddings to perform the task of edge-sign prediction. Even though it is still has the same learning parameters (*Emb*_*sign*_) as the **TEA**, it is neither using the Structural Balance Theory nor is it using the higher order structures. While we do not report the results for this case, we notice that the model's performance falls down drastically. It is just slightly better than the performance of the N2V model and worse than many of the considered baselines. The AUC is (0.77, 0.70 and 0.656) and the Micro-F1 score is (0.904, 0.811, 0.885) for Epinions, Slashdot and Wiki datasets respectively.

This suggests that even a vanilla edge-sign classification model benefits drastically by incorporating the Structural Balance Theory and higher order structures.

### 5.6. Case Study

In this section, we perform a case study to understand how sampling multiple triads and using attention mechanism augments the use of Structural Balance Theory in our model.

Consider the edge between nodes 7, 406 and 22, 295 in the Epinions dataset. If we just use the *Emb*_*sign*_ model and do not sample any triads, the model predicts the edge sign to be negative even though the correct edge sign is positive. Now if we sample triads and use them along with the *Emb*_*sign*_ model, the predicted sign becomes positive which is the correct sign. There are two triads in the graph corresponding to this edge and both the triads have the sign “pos-pos.” In such a setting, sampling triads is sufficient to improve the performance of the model. Next, we will take an example to show why attention mechanism is needed.

Consider the edge between nodes 966 and 7, 588 in the Epinions dataset. The true sign for the edge is positive. When we use the **TE** model (where we sample multiple triads but do not use attention mechanism), the model predicts the sign to be negative with high confidence. The reason for this observation is that there are 30 triads for the given edge. 22 of these triads have the sign “neg-pos” and just 8 triads have the sign “pos-pos.” Given this imbalance, The “neg-pos” signs are likely to be in majority among the sampled triads. We can not counter this problem by sampling more triads alone. Our **TEA** model can handle such cases easily. When we use the **TEA** model, the model predicts the edge sign to be positive by giving a higher attention score to the “pos-pos” triads (even though they are fewer) and less attention weight to the “neg-pos” triads. This redistribution of importance by means of attention scores helps to complement the strenghts of the Structural Balance Theory.

## 6. Conclusions

This paper studied network embedding for the signed network, and an approach called **TEA** was proposed. **TEA** learned network embedding by predicting the sign of edges in a network. Inspired by the Structural Balance Theory, for each edge between two nodes, **TEA** predicted the edge sign by considering different triads connecting the two nodes. Moreover, an attention mechanism was leverage to weighted integrate the information from different triads during prediction. Experimental results on real-world signed networks prove the effectiveness of **TEA**. In the future, we plan to study network data with more edge types, such as the heterogeneous networks (Sun and Han, 2013) and the knowledge graphs (Bordes et al., 2013).

## Author Contributions

SS and MQ implemented the model, ran the experiments and prepared the manuscript. JT guided the entire effort and helped with the ideation and brainstorming and finalizing the manuscript.

### Conflict of Interest Statement

The authors declare that the research was conducted in the absence of any commercial or financial relationships that could be construed as a potential conflict of interest.

## Footnotes

^1^http://snap.stanford.edu/data/soc-sign-epinions.html

^2^http://snap.stanford.edu/data/soc-sign-Slashdot090221.html

^3^Available at http://snap.stanford.edu/data/#signnets
